# Exploring the role of palmitoylation in sepsis: mechanistic insights and future perspectives

**DOI:** 10.1186/s10020-025-01284-5

**Published:** 2025-06-03

**Authors:** Susu Cao, Wenyan Xiao, Sinong Pan, Tianfeng Hua, Min Yang

**Affiliations:** 1 The Second Department of Critical Care Medicine, The Second Affiliated Hospital of Anhui Medical University, 678 Furong Road, Hefei, 230601 Anhui Province China; 2 Laboratory of Cardiopulmonary Resuscitation and Critical Care, The Second Affiliated Hospital of Anhui Medical University, 678 Furong Road, Hefei, 230601 Anhui Province China

**Keywords:** Sepsis, Palmitoylation, Immunity, Pathogenesis

## Abstract

The palmitoylation system is intricate, multidimensional, and plays a crucial role in various inflammatory and immune-related disorders. Palmitoylation controls protein stability, cargo sorting, signal transmission, as well as cell differentiation and death. Notably, a growing body of studies has highlighted its participation in inflammatory processes, either directly or indirectly, indicating its broad and complex involvement in the development of sepsis. Understanding the mechanisms underlying palmitoylation is essential for advancing research on sepsis. We began this review with a brief summary of research related to sepsis progression. Second, we went over recent studies on palmitoylation. Third, we compiled and described palmitoylation-related alterations in vital molecules or biological processes involved in sepsis. Lastly, we outlined the promising features of palmitoylation and proposed a hopeful outlook for future research in sepsis.

## Introduction

Sepsis is a life-threatening disease, that was described as early as 1600 B.C. in Egyptian scrolls. It currently poses a serious threat to world health, especially in low-income regions such as sub-Saharan Africa, Oceania, South Asia, East Asia, and Southeast Asia (Rudd et al. 2020). According to a 2017 report by the Institute for Health Metrics and Evaluation (IHME), over 48.9 million sepsis cases occurred worldwide (Rudd et al. 2020). The mortality rate among patients admitted to intensive care units (ICUs) ranges from 24.3 to 60.0%, influenced by a number of parameters such as age, comorbidities, and pathogen type (Fleischmann et al. 2016; Ferrario et al. 2016; McPherson et al. 2001; Georgescu et al. 2017; Huang et al. 2016). Sepsis was designated a global health priority by the World Health Organization (WHO) in 2017, emphasizing the need for better prevention, recognition, and treatment (Fleischmann 2016). Sepsis is characterized by hyperinflammatory response, marked by the excessive release of cytokines such as interleukin (IL)−1β, IL-6, and tumor necrosis factor-α (TNF-α), as well as multiple organ dysfunction syndrome (MODS), which affects the heart, liver, kidneys, lungs, spleen, and brain (Lelubre and Vincent 2018). Additionally, numerous studies have been conducted on inflammation activation in the early stages of sepsis as well as immune paralysis in the later stages. Researchers are seeking novel therapeutic strategies based on underlying mechanisms. Notably, new studies have brought attention to the significance of various post-translational modifications (PTMs) of sepsis-related key regulators, which can alter protein activity—the final executor of diverse biological processes. Among various PTMs, palmitoylation—a reversible lipid modification—has also gained increasing attention in sepsis research.

Palmitoylation plays a crucial role in intercellular signal transduction, protein localization, and functional regulation. Its complexity is increased by its reversible nature, which permits dynamic regulation. Palmitate, a 16-carbon saturated fatty acid (palmitic acid, PA), undergoes palmitoylation when covalently attached to a cysteine (Cys) residue via a thioester bond (Linder and Deschenes 2007). Palmitate is synthesized de novo from fatty acids by the enzyme fatty acid synthase (FASN) (Kim et al. 2019). Palmitoylation is catalyzed by the zinc-finger DHHC-type containing (ZDHHC) protein family, which includes ZDHHC1-ZDHHC9 and ZDHHC11-ZDHHC24 (Abrami et al. 2008; Wang et al. 2024). Conversely, depalmitoylation is mediated by acyl-protein thioesterases (APT1/2), palmitoyl protein thioesterases (PPT1/2), and α/β hydrolase domain-containing proteins 17 A/B/C (ABHD17 A/B/C) (Ko and Dixon 2018). Theoretically, enzymes involved in palmitoylation and depalmitoylation regulate the palmitoylation status of key molecules, thereby modulating the pathological immune environment and influencing disease initiation and progression. And enzyme-targeted therapeutics hold enormous potential to improve disease outcomes and offer novel treatment strategies. Thus, comprehensive understanding of palmitoylation in sepsis is critically important**.**

Recent studies have increasingly highlighted the close relationship between palmitoylation and sepsis, particularly in terms of its function as a sepsis-associated modifier. In order to construct a thorough framework that elucidates palmitoylation’s impact on sepsis progression and offers fresh perspectives for future research and therapeutic strategies, this review examines current findings on the interplay between palmitoylation and sepsis. First, we provided a general overview of sepsis, covering its definition, pathogenesis, and associated treatments. Second, we concisely discussed the definition, function, regulation, emerging detection methods, and therapeutic strategies related to palmitoylation and depalmitoylation. Third, we thoroughly reviewed and summarized mechanisms of sepsis from the perspective of palmitoylation, with a particular focus on its role in key aspects of sepsis progression. Fourth, we proposed potential palmitoylation-associated characteristics in sepsis and discussed their promising therapeutic implications. Additionally, we addressed future challenges in investigating sepsis-related palmitoylation modifications.

### The overview of sepsis-mediated immunity

Unlike a simple infection, sepsis is not merely defined by the presence of pathogens. According to the latest Sepsis-3.0 definition, sepsis is a complex pathophysiological syndrome involving three key elements: infection, a dysregulated host response, and MODS (Singer et al. 2016). Previously, sepsis diagnosis primarily relied on the Systemic Inflammatory Response Syndrome (SIRS) criteria, which overemphasized inflammatory reactions. Moreover, early perspectives on sepsis-related immunity mostly focused on the superiority of innate immunity over adaptive immunity (Nedeva 2021). The current paradigm has shifted to acknowledge the early activation of both pro- and anti-inflammatory pathways and suggests that innate and adaptive immunity may occur synchronously, highlighting the complexity of the immunological milieu in sepsis (Delano and Ward 2016; Jacobi 2022). Additionally, increasing attention has been paid to non-immune alterations (such as cardiovascular, neurological, hormonal, bioenergetic, metabolic, and coagulation dysregulation) that collectively contribute to pathogenesis (Singer et al. 2016). Clinically, patients with suspected infection and a SOFA score of 2 or more can be diagnosed with sepsis (Singer et al. 2016).

Pattern recognition receptors (PRRs) are essential immune sensors primarily expressed on innate immune cells, including dendritic cells (DCs), macrophages, neutrophils, and on B cells of the adaptive immune system. PRRs detect pathogen-associated molecular patterns (PAMPs) and damage-associated molecular patterns (DAMPs), triggering immune responses (Kawai and Akira 2011; van der Poll et al. 2017). Major subclasses include Toll-like receptors (TLRs), AIM2-like receptors (ALRs), nucleotide oligomerization domain leucine-rich proteins (NLRs), C-type lectin receptors (CLRs), and RIG-I-like receptors (RLRs), reflecting the pleiotropy and complexity of immunological signaling (Kawai and Akira 2011). However, an excessive pathogen burden or immunodeficiency can dysregulate the immune response, exacerbating tissue damage (Kawai and Akira 2011). Upon activation by DAMPs and PAMPs, PRRs trigger cytokine cascades, leading to the production of IL-1β, IL-18 (Pålsson-McDermott et al. 2004; Pålsson-McDermott et al. 2004; Xiang 2015), as well as IL-6, IL-8, TNF-ɑ, and IL-17 (Liu et al. 2019; Morrow 2019). These cytokines collectively contribute to the “cytokine storm”, a critical contributor in multi-organ damage and early sepsis mortality (Chousterman et al. 2017). On the other hand, anti-inflammatory cytokines such as IL-10, IL-27, and IL-33 counteract the “cytokine storm”, aiming to restore immune homeostasis (Morrow 2019). IL-38 guards against sepsis by inhibiting CD8^+^T cell apoptosis and NOD-like receptor protein 3 (NLRP3) activation (Ge 2021). Given that the production and secretion of inflammatory cytokines initiate immune activation, the central role of cellular immunomodulation in sepsis has garnered increasing attention. Neutrophils are the first to migrate rapidly to the infected sites, eliminating pathogens through phagocytosis, cytokine release, and reactive oxygen species (ROS) production (Valeria 2021). Meanwhile, mature neutrophils increase at disease onset, characterized by degranulation and protease release (Martín-Fernández et al. 2021). Additionally, pathogens are efficiently captured by neutrophil extracellular traps (NETs), which are made up of histones, myeloperoxidase, and elastase (Ravindran et al. 2019). Notably, macrophages-derived microvesicles can accelerate the formation of NETs through Gasdermin D (GSDMD)-N-expressing mitochondrial transfer (Kuang et al. 2024). Depending on the tissue setting, monocyte-derived macrophages can differentiate into distinct phenotypes (M1/M2) in order to preserve inflammation balance. Natural killer (NK) cells coordinate with macrophages through IFN-γ, mediating cell necrosis and systemic inflammation (Guo et al. 2018). Lymphocytes interact with innate immune cells such as monocytes, neutrophils, and DCs through divergent differentiation routes, partly mediated by cytokines (Tham et al. 2002; Xue et al. 2019; Xue et al. 2019). However, in the late-stage of sepsis, lymphocytopenia and low HLA-DR expression are both associated with poor prognosis (Quirant-Sánchez et al. 2023). Analogously, HLA-DR^low^monocytes are linked to poor survival, which could be identified by single-cell RNA sequencing (scRNA-seq), which reveals an immunosuppressive monocyte subclass (Quirant-Sánchez et al. 2023; Yao 2023). Other immunosuppressive cells, such as regulatory T cells (Tregs), are significantly upregulated in late-stage sepsis, suppressing T and B cell activation. Both NETs and highly expressed PD-L1 can stimulate their proliferation and differentiation from CD4^+^T cells (Shi 2024; Coman O et al. 2024; Wang et al. 2021). Recently, the PD-1/PD-L1 axis has been recognized as a central modulator of sepsis-induced immunosuppression. PD-1 upregulation in T cells promotes apoptosis, while PD-L1 expression in neutrophils delays apoptosis via PI3 K/AKT pathway, thereby exacerbating acute lung injury (ALI) (Coman et al. 2024; Wang et al. 2021). Additionally, it is known that IL-10 and IL-33 are key mediators of immune paralysis (Liu et al. 2019; Morrow 2019; Xu et al. 2022). IL-6 exerts dual effects: it restricts Treg proliferation while inducing IL-10, which in turn suppresses IL-17, a cytokine critical for T helper 17 (Th17) cell differentiation (McGeachy et al. 2007). These dynamic immuno-inflammatory fluctuations highlight the need for a deeper understanding of the molecular mechanisms governing immune activation and regulation.

As previously mentioned, PAMPs and DAMPs are triggered by various stimuli, including microbial products, host glycoproteins, lipoproteins, and nucleic acids (Kawai and Akira 2011). Upon sensing these signals, PRRs activate a cascade of intracellular signaling pathways. The major component of the bacterial cell wall, lipopolysaccharides (LPS), are recognized by TLR4, which then activates IKK complex in a MyD88-dependent manner. Following this, activated NF-κB translocates into the nucleus and induces the production of proinflammatory cytokines, including IL-6, TNF-α, pro-IL-1β, and pro-IL-18. Activation of the NLRP3 inflammasome, a protein complex under intensive investigation, leads to caspase-1-mediated cleavage of pro-IL-1β and pro-IL-18 into IL-1β and IL-18 (Hafner-Bratkovič and Pelegrín 2018). Furthermore, it cleaves GSDMD to produce GSDMD-N terminal fragment (GSDMD-NT), a crucial executor of pyroptosis. GSDMD-NT promotes this pro-inflammatory form of cell death by creating membrane pores (Gong et al. 2018; Fu and Wu 2023). Moreover, when double-stranded DNA from virus is detected by cyclic GMP-AMP synthase (cGAS), it activates the stimulator of interferon genes (STING) pathway, leading to interferon regulatory factor 3 (IRF3) phosphorylation, type I IFN production, and NLRP3 activation (Burdette 2011). The sepsis-microenvironment, at the single-cell level, is also highly systemic and complex. Moreover, intracellular signal interactions play a critical role in sepsis progression. Notably, in addition to direct interactions, vesicle-mediated signaling plays a key role in both promoting and inhibiting sepsis and is under intensive explorations (Iba et al. 2022; Xie et al. 2023). In this context, disruption of TLR sensing or conformational activation of NLRP3, GSDMD, STING, or other related pathways may influence sepsis progression.

In addition to conventional treatments such as early detection, antibiotic therapy, fluid resuscitation, and vasoactive agents in the early stages of sepsis, researchers have proposed alternative therapies based on the pathobiological background. Glucocorticoids can decrease inflammation to some extent. However, its administration remains controversial. Some studies have shown that it lowers 28-day mortality (Fang et al. 2019). However, its potential risks, such as hyperlactatemia and lethal shock, suggest that it should be used cautiously in clinical practice (Vandewalle et al. 2021). Additionally, novel drugs targeting key molecules and aiming to reduce MODS are being investigated. For example, norwogonin has been shown to attenuate sepsis-induced ALI, while fraxetin alleviated spleen injury in sepsis (Cao et al. 2025; Huangfu et al. 2025). In the late stages of sepsis, it is crucial to monitor and dynamically adjust immune status, focusing on immune response stimulation and the replacement of immunological factors, such as intravenous immunoglobulins. However, different immunoglobulin preparations in septic patients exhibit distinct opsonic and protective activities (Rossmann et al. 2015). Other treatments, including immunostimulatory cytokines, negative costimulatory molecule antibodies and inhibitors, have also been considered for sepsis treatment, although their efficacy requires further validation. In conclusion, precision medicine for sepsis requires a comprehensive understanding of its mechanisms and heterogeneous nature.

### The overview of palmitoylation

Palmitoylation is a widespread and intricate protein modification process, involved in membrane localization, signaling activation, protein stability, and vesicle release and transportation in cancer, inflammatory diseases, and other pathological conditions (Chamberlain and Shipston 2015). The addition of palmitate to specific or multiple cysteine residues induces protein changes, which subsequently affect downstream functions and molecules (Chamberlain and Shipston 2015).

The key enzymes catalyzing palmitoylation and depalmitoylation include palmitoyl-acyl transferases (PATs), APT1/2, PPT1/2, and ABDHs, which together form a regulatory network. A cascade interaction has been observed between human ZDHHC6 and ZDHHC16. Researchers found that ZDHHC6 is regulated by its upstream enzyme ZDHHC16 through modification at Cys328, Cys329, and Cys343. The interaction between ZDHHC16 and APT2 enables cells to fine-tune ZDHHC6 activity, and this interaction varies across species (Abrami et al. 2017). Adding to the complexity of this regulatory network, APT1/2 and ABDH-family thioesterases are also palmitoylated to ensure proper membrane localization and function (Yang et al. 2010; Kong et al. 2013). Additionally, palmitoyltransferases themselves undergo phosphorylation, further contributing to the regulatory networks (Lievens et al. 2016). Substrate selection by ZDHHC enzymes does not follow a strict one-to-one relationship. Instead, specific ZDHHC enzymes have stronger effect on the certain substrates than others (Ohno et al. 2012). Different ZDHHC enzymes also show distinct cellular distributions. Most ZDHHC proteins are localized to the endoplasmic reticulum (ER) or Golgi apparatus, while ZDHHC5, ZDHHC20, and ZDHHC21 are mainly found on the plasma membrane (Ohno et al. 2006). Interestingly, in 2017, researchers proposed that this process is generic and stochastic, based on native mass spectrometry (MS) findings. They further suggested that this modification is determined by the accessibility of PATs to cysteine residues on membrane-embedded proteins (Rodenburg et al. 2017). Dynamic changes in regulators involved in palmitoylation and depalmitoylation appear to be essential for maintaining cellular functions.

**Fig. 1:**
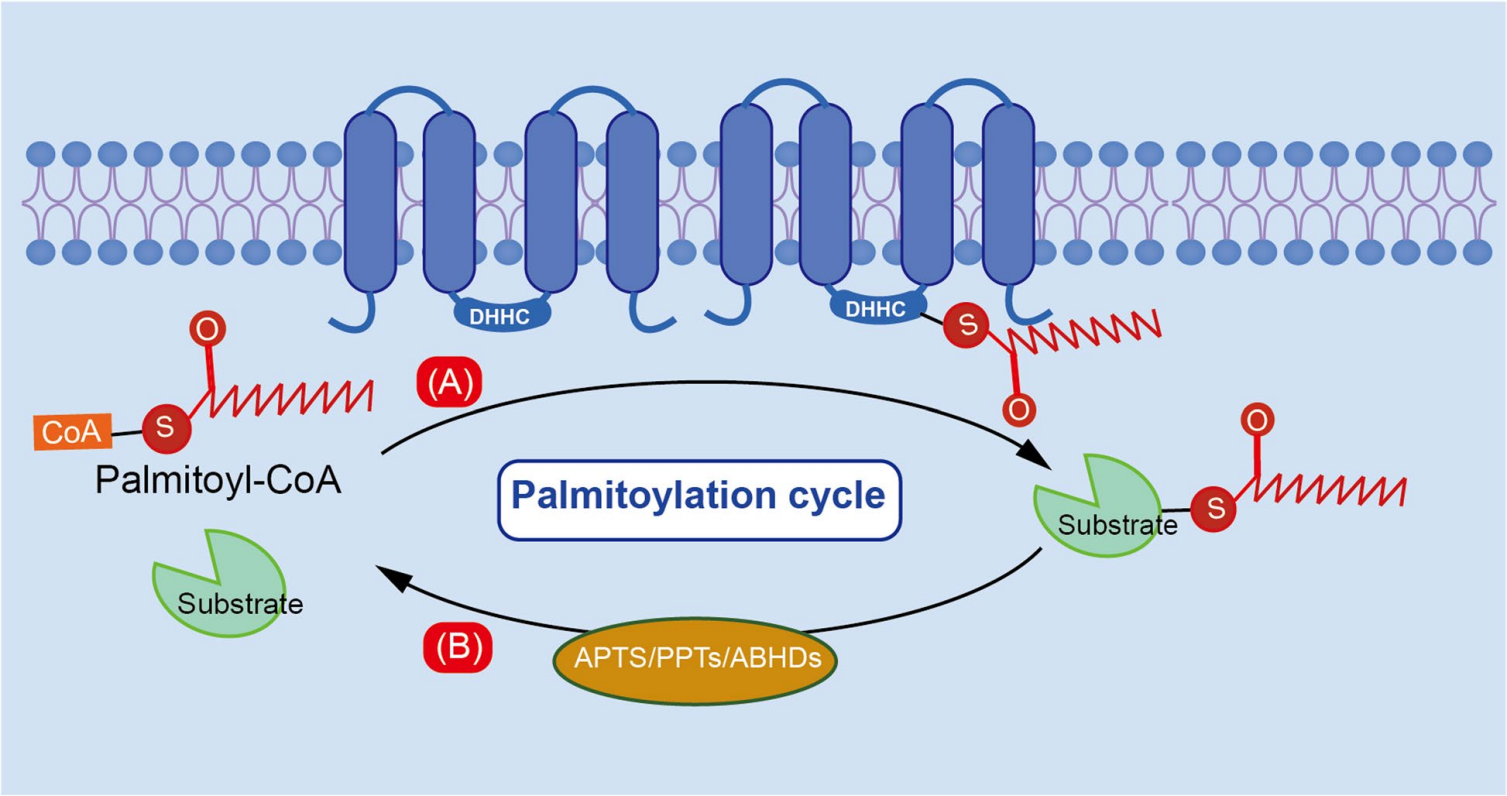
Schematic overiew of the palitoylation and depalmitoylation processes. **A** Palmitoylation involves two sequential steps: (1) Auto-palmitoylation—the palmitoyl group is transferred from palmitoyl-CoA to the active site cysteine of the palmitoylating enzyme, forming a thioester bond; (2) Substrate palmitoylation-the palmitoylated enzyme subsequently transfers the palmitoyl group to the target protein. **B** Depalmitoylation is primarily mediated by APTs, PPTs, and ABHDs, which hydrolyze the thioester bond and remove the palmitoy group from substrates. Abbreviations: APTs, acyl-protein thioesterases; ABHDs,α/βhydrolase domain-containing proteins PPTs, palmitoyl protein thioesterases

Early studies have demonstrated that palmitoylation plays a crucial role in cargo transport between intracellular organelles, including mitochondria, lysosomes, the Golgi apparatus, and the ER (Rudnik 2022; Ernst et al. 2018; Liu et al. 2024). Additionally, it can mediate the specific localization of certain molecules within cells. For example, palmitoylation facilitates the transport of non-transmembrane proteins to specific neuronal compartments, such as newly formed axons (Tortosa and Hoogenraad 2018). The regulation of palmitoylation turnover is essential for normal biological processes. It also alters the distribution and expression of membrane receptors, thereby modulating signal transduction through receptor-ligand interactions. In addition, the structural integrity of some proteins partially depends on their palmitoylation modifications (Murphy and Kolandaivelu 2016; Zhou et al. 2023). Beyond its canonical role in protein and membrane regulation, palmitoylation can influence the pathogenic potential of malignant cells by modifying key oncogenes, such as the MYC gene in pancreatic cancer (Zhang et al. 2024), the RAS gene in lung cancer (Kharbanda 2020), and multiple genes in hematopoietic malignancies (Yu and Qian 2023; Zhao et al. 2015; Cuiffo and Ren 2010). In coordination, other lipid-modification-related genes also regulate the initiation and progression of malignancies, offering potential therapeutic approaches for cancer treatment (Bu et al. 2024). Additionally, researchers have attempted to design effective cancer treatment strategies based on palmitoylation-mediated drug-resistance and anti-tumor immunity (Sun et al. 2022; Sun et al. 2023; Fan et al. 2023). Literature indicates that in malignant diseases, depalmitoylation is usually associated with improved prognosis, while palmitoylation tends to worsen malignancy (Bu et al. 2024; Pei et al. 2022; Sun et al. 2022; Sun et al. 2023; Fan et al. 2023). Conversely, in inflammatory diseases, palmitoylation plays a complex, double-edged role. Non-alcoholic steatohepatitis (NASH) is closely associated with inflammatory events. Disrupted free fatty acid (FFA) metabolism may mediate this process through CD36 palmitoylation (Zhao et al. 2018). Palmitoylation-mediated Th17 differentiation also promotes the development of inflammatory bowel diseases (IBD) (Zhang et al. 2020). However, palmitoylation enhances chaperone-mediated autophagy (CMA), decreasing NLRP3 activation and preventing a sustained inflammation (Wang and Cui 2023). Interestingly, NLRP3 transmission and downstream GSDMD-mediated cytokine secretion also partially depend on specific palmitoylation modification. Figure 1 exhibits the processes of palmitoylation and depalmitoylation. In conclusion, a deeper understanding of this complex regulatory network and the underlying mechanisms in both physiological and pathological conditions is essential for developing more effective treatments. Above all, it is crucial to develop and apply more sensitive and specific techniques to measure palmitoylation level in cells or tissues.

In the early twentieth century, researchers used radioisotopic labeling to identify palmitoylation events (Jochen and Hays 1993). However, the labeling site and degree could affect the accuracy of results, and the use of radionuclides posed health risks to researchers. Currently, acyl-biotin exchange (ABE) and click chemistry are commonly used to detect palmitoylation events (Wei et al. 2024; Brigidi and Bamji 2013). However, false-positive results may still occur. To comprehensively identify palmitoylated substrates, MS plays a critical role. MS enables the identification and enrichment of all substrates associated with specific palmitoyltransferases. This approach offers promising insights and greatly facilitates further research (Wang and Schey 2018).

Nowadays, researchers are exploring novel drugs related to palmitoylation in both fundamental research and clinical studies. Notably, 2-Bromopalmitate (2-BP) is commonly utilized in research to inhibit DHHC acyltransferase activation. Due to its anti-cancer effects, 2-BP has attracted growing research interest. For example, 2-BP can reduce chemotherapy-induced cell pyroptosis by inhibiting Gasdermin E (GSDME) palmitoylation (Hu 2024). Moreover, it promotes Th cell expansion, facilitates CD8^+^cytotoxic T cell activation, and enhances tumor cell killing (Xin et al. 2025). Furthermore, combining 2-BP with bionic nanodrug therapy provides a novel and promising approach to cancer treatment (Liu et al. 2024; Tan 2023). In addition, 2-BP injection in mice alters the gut microbiota composition (Ma 2024). Serum PA is also linked to cardiovascular dysfunction by accelerating endothelial injury, which can be mitigated by 2-BP (He et al. 2025). Researchers also discovered that 2-BP inhibits viral replication by depleting lipid droplets, highlighting its potential in drug development (Liu et al. 2024). Similarly, APT-1/2 inhibitors have also been studied. Palmostatin B, an APT-1/2 inhibitor, shows potential in treating melanoma by targeting the palmitoylation of the NRAS oncogene (Vujic et al. 2016). Given the development of palmitoylation-related drugs, palmitoylation shows great potential in treating cancer, inflammatory diseases, and infections.

### Sepsis-related S-palmitoylation

Given the critical importance of PTMs in sepsis and the diverse biological functions of palmitoylation, this section explores how palmitoylation regulates TLR signaling, NLRP3 inflammasome assembly, GSDMD translocation and oligomerization, STING pathway activation, and other mechanisms contributing to sepsis development (Table 1). It aims to summarize the complex and multifaceted roles of palmitoylation in sepsis, as well as its potential therapeutic implications.

**Table 1:** Key regulators involved in sepsis and their association with palmitoylation

Sepsis-related regulators	Function	Palmitoylation-effect	Associated DHHC enzymes
TLR	Pathogen recognition	Sensor activity; Membrane expression; Lipid rafts localization	ZDHHC2/6/7/15 (Kim et al. 2019; Borzęcka-Solarz et al. 2017; Zhang et al. 2021)
NLRP3	Pro-inflammation	Translocation; Oligomerization	ZDHHC1/5/7/12/17 (Zheng et al. 2023; Lu et al. 2019; Nie et al. 2024)
GSDMD	Pro-pyroptosis	Activity; Membrane localization	ZDHHC5/7/9/14 (Zhang et al. 2024; Du et al. 2024; Zhuang et al. 2024)
STING	Type I interferon activation	Sensor activity	ZDHHC18 (Shi et al. 2022)
Others (EVs; α1 AR; CD36 complex)	Vesicular trafficking; Receptor responsiveness; Damage resolution	Signal transduction; Drug responsiveness; Formation	ZDHHC21 (Xie et al. 2021; Yang et al. 2021); ZDHHC18/21 (Marin et al. 2016);/

#### TLRs

**Fig. 2:**
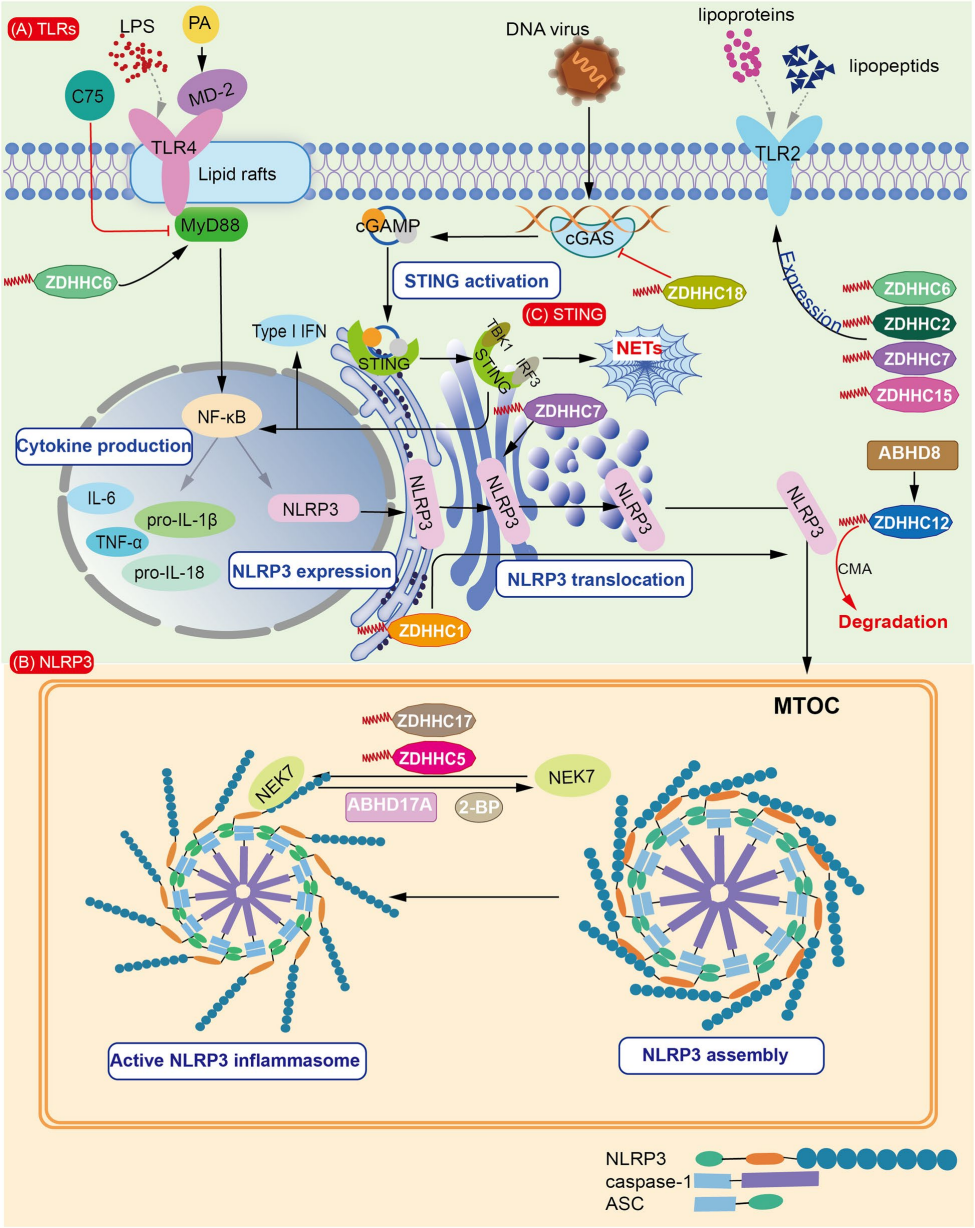
Relationships between palmitoylation and sepsis-related key factors (TLRs, NLRP3, STING). **A** TLR4 senses signals from LPS, a process enhanced by serval factors. PA and ZDHHC6 promote TLR4 activation by strengthening its interaction with MD-2 and facilitating MyD88-depedent signal transduction, respectively (Park et al. 2009; Nicholas et al. 2017; Wang 2018). C75, a fatty acid synthase inhibitor, suppresses MyD88-depedent signaling (Kim et al. 2019). Palmitoylation increases the localization of TLR4 within lipid rafts (Sapo 2023). TLR2, another major receptor for lipoproteins and lipopeptids, exhibits elevated membrane expression via palmitoylation mediated by ZDHHC2, ZDHHC6, ZDHHC7, and ZDHHC15 (Borzęcka-Solarz et al. 2017; Chesarino NM et al. 2014; Zhang et al. 2021). **B **Upon activation of NF-κB, the expression of IL-6, TNF-α, pro-IL-1β, and pro-IL-18 is upregulated, contributing to inflammation. NLRP3 expression is also increased via NF-κB signaling. ZDHHC1 regulates the translocation of NLRP3 from the ER to the dTGN, and finally to the MTOC (Nie et al. 2024). ZDHHC7 stabilizes NLRP3 at the resting TGN (Yu et al. 2024). NLRP3 undergoes oligomerization and assembly at the MTOC. During this process, ZDHHC5 and ZDHHC17 enhance the interaction between NEK7 and NLRP3, which can be reversed by ABHD17 A and 2-BP (Zheng et al. 2023; Hu et al. 2024). ABHD proteins also exert inhibitive regulatory effects on NLRP3 through ZDHHC12-mediated palmitoylation and CMA-dependent degradation (Yang et al. n.d.). **C **cGAS detects cytosolic double-stranded DNA and subsequently generates cGAMP to activate the STING pathway. This sensing process can be inhibited by ZDHHC18 (Shi et al. 2022). Abbreviations: 2-BP, 2-Bromopalmitate; ABHD, α/β hydrolase domain-containing protein; cGAS, cyclic GMP-AMP synthase; cGAMP, cyclic GMP-AMP; CMA, chaperone-mediated autophagy; dTGN, dispersed trans-Golgi network; ER, endoplasmic reticulum; IRF3, interferon regulatory factor 3; MD-2, myeloid differentiation protein 2; MTOC, microtubule-organizing center; NETs, neutrophil extracellular traps; NLRP3, NOD-like receptor protein 3; PA, palmitic acid; STING, stimulator of interferon genes; TBK1, TANK-binding kinase 1; TLRs, Toll-like receptors

TLRs play a central role in pathogen recognition and are critical for initiating innate immunity in both bacterial and viral sepsis. Proper TLR function requires palmitoylation of specific amino acid residue to ensure correct protein folding, localization, and activity. Notably, TLR4 primarily recognizes LPS, a key component of Gram-negative bacterial cell walls (Poltorak et al. 1998). Myeloid differentiation protein-2 (MD-2) is an accessory protein that facilitates LPS recognition by directly binding to it (Park et al. 2009). PA has been shown to stimulate the canonical NF-κB pathway via TLR4 (Nicholas et al. 2017). Mechanistically, PA directly interacts with MD-2 in an inflammasome-dependent manner, promoting ROS generation, TLR4 activation, and IL-1β production in human DCs, thereby exerting its maximum immune-stimulatory effects (Nicholas et al. 2017). Similarly, PA can induce inflammatory injury in myocardiocytes via TLR4 activation pathway, providing insights into the mechanisms of obesity-associated myocardiocyte damage (Wang 2018). In addition to TLR4 pathway regulation by PA with MD-2 in sepsis, metabolism-mediated immune reprogramming by PA is increasingly recognized. A 2022 study suggested that PA may increase susceptibility to sepsis by reprogramming innate immunity through dietary influences, implying dynamic changes in palmitoylation levels within the septic microenvironment (Seufert et al. 2022). However, the detailed relationship between nutrition and immune metabolism remains unclear. Besides PA/MD2 modification, MyD88-an essential TLR adaptor linked to downstream NF-κB activation-can also be regulated by S-palmitoylation. The fatty acid synthase inhibitor (C75) improves survival in mice with CLP mouse models by blocking TLR/MyD88 signaling in neutrophils (Kim et al. 2019). Mechanistically, ZDHHC6 palmitoylates MyD88 at Cys113, promoting the recruitment of interleukin-1 receptor-associated kinase 4 (IRAK4) to the Myddosome complex, thereby activating NF-κB in septic mouse models (Kim et al. 2019). Lipid rafts are membrane microdomains rich in cholesterol and sphingolipids, playing a role in vesicle formation (Kim et al. 2019). TLR4 tends to accumulate in these earas, with palmitoylated Lyn essential for this process, thus promoting signal transduction (Sapo 2023). Notably, TLRs may also serve as direct ligands of ZDHHCs. Researchers reported that in DCs, palmitoylation by ZDHHC2, ZDHHC6, ZDHHC7, and ZDHHC15 is pivotal for enhancing TLR2 surface expression, which recognizes bacterial lipoproteins and lipopeptids (Chesarino et al. 2014; Zhang et al. 2021; Oliveira-Nascimento et al. 2012). Overall, palmitoylation potentially affects TLR accessory proteins, adaptors, translocation, and membrane expression. Interaction among diverse TLR-associated proteins, amino acid composition across TLR domains, and their palmitoylation status may collectively lead to phenotypic changes in sepsis. A comprehensive understanding of palmitoylation and depalmitoylation in sepsis-encompassing the net effects of these regulations, including enzymes, TLR types, binding partners, and distinct reaction conditions-is still lacking. Furthermore, how genomic profiles, metabolic phenotypes, and clinical subtypes differentially regulate TLR palmitoylation in sepsis requires further study and clinical translation. The influences about the palmitoylation of TLRs in sepsis is presented in Fig. 2.

#### NLRP3

NLRP3, a member of NOD-like receptor (NLR) family, is a type of PRRs found in cytoplasma that forms the NLRP3 inflammasome, a key complex sepsis development. The NLRP3 inflammasome consists of NLRP3, apoptosis-associated speck-like protein containing a CARD (ASC) and procaspase-1 (Fu and Wu 2023). Notably, NLRP3 inflammasome assembly is a key step in inducing cell apoptosis, primarily by cleaving GSDMD into GSDMD-NT (Gong et al. 2018; Fu and Wu 2023). At the same time, the release of mature cytokines further promotes inflammation (Hafner-Bratkovič and Pelegrín 2018). The microtubule-organizing center (MTOC), which includes the centrosome and basal body, plays a crucial role in organizing microtubules, facilitating molecular motor activity, and directing transport and polarization of subcellular components (Kloc et al. 2014). NEK7 is a crucial component of the NLRP3 inflammasome. Downregulating NEK7 can alleviate pyroptosis in sepsis-induced acute kidney injury (Zhang et al. 2022). Mechanisitically, NLRP3 oligomerization and its interaction with NEK7 promote inflammasom conformation (Yang et al. 2020). The MTOC is regarded as the final site for NLRP3 assembly (Kloc et al. 2014; Yang et al. 2020; Li 2017). ZDHHC5 and ZDHHC17 have been reported to enhance NEK7-NLRP3 interactions at Cys837/838 and Cys419 residue, respectively, which can be reversed by ABHD17 A and 2-BP (Zheng et al. 2023; Hu et al. 2024)In contrast, ZDHHC12-mediated termination effect on NLRP3 was reported (Yang et al. n.d.). ABHD8, a member of the ABHD family, acts as a scaffold to recruit ZDHHC12-mediated NLRP3 palmitoylation and subsequent CMA-mediated degradation, thereby preventing inflammasome activation (Yang et al. n.d.). Furthermore, ABHD8 overexpression ameliorates LPS-triggered inflammation in vivo, offering therapeutic insights for sepsis (Yang et al. n.d.). Similarily, vaccarin could act on ZDHHC12 to induce NLRP3 palmitoylation to inactivate it, which is blocked by NLRP3 agonist ATP and 2-BP (Zhu et al. 2024). Recently, the versatile regulatory role of ZDHHC7 in palmitoylating NLRP3 at Cys126 has encouraged septic explorations (Yu et al. 2024). Mechanistically, ZDHHC7-mediated palmitoylation promotes NLRP3 localization at the resting trans-Golgi network (TGN) and activates it at the dispersed TGN, which is important for ASC recrutiment and assembly (Yu et al. 2024). Additionally, ZDHHC5 has been reported to assist in the membrane recruitment and immune signaling of sepsis-related receptors (Lu et al. 2019). Collectively, inflammasome palmitoylation is multi-layered and warrants coordination among various organelles (Pandey et al. 2021). In addition to localization at Cys126, palmitoylation at Cys898 facilitates NLRP3 translocation to dispersed TGN (dTGN) vesicles (Leishman et al. 2024). Interestingly, a more detailed trafficking route of NLRP3 inflammasome activation has been described. ZDHHC1-mediated palmitoylation at Cys130 and Cys985 promotes NLRP3 trafficking among subcellular membranes, including mitochondria, dTGN, and endosomes (Nie et al. 2024). Ultimately, all this dynamic trafficking converges at the MTOC. In summary, palmitoylation orchestrates NLRP3 at multiple layers, including translocation, assembly, and activation under LPS stimulation. However, the effects of palmitoylation-assisted NLRP3 on sepsis-mediated immunity and inflammation across organs at stages remain unclear. The palmitoylation impacts on NLRP3 in sepsis is exhibited in Fig. 2.

#### GSDMD

**Fig. 3:**
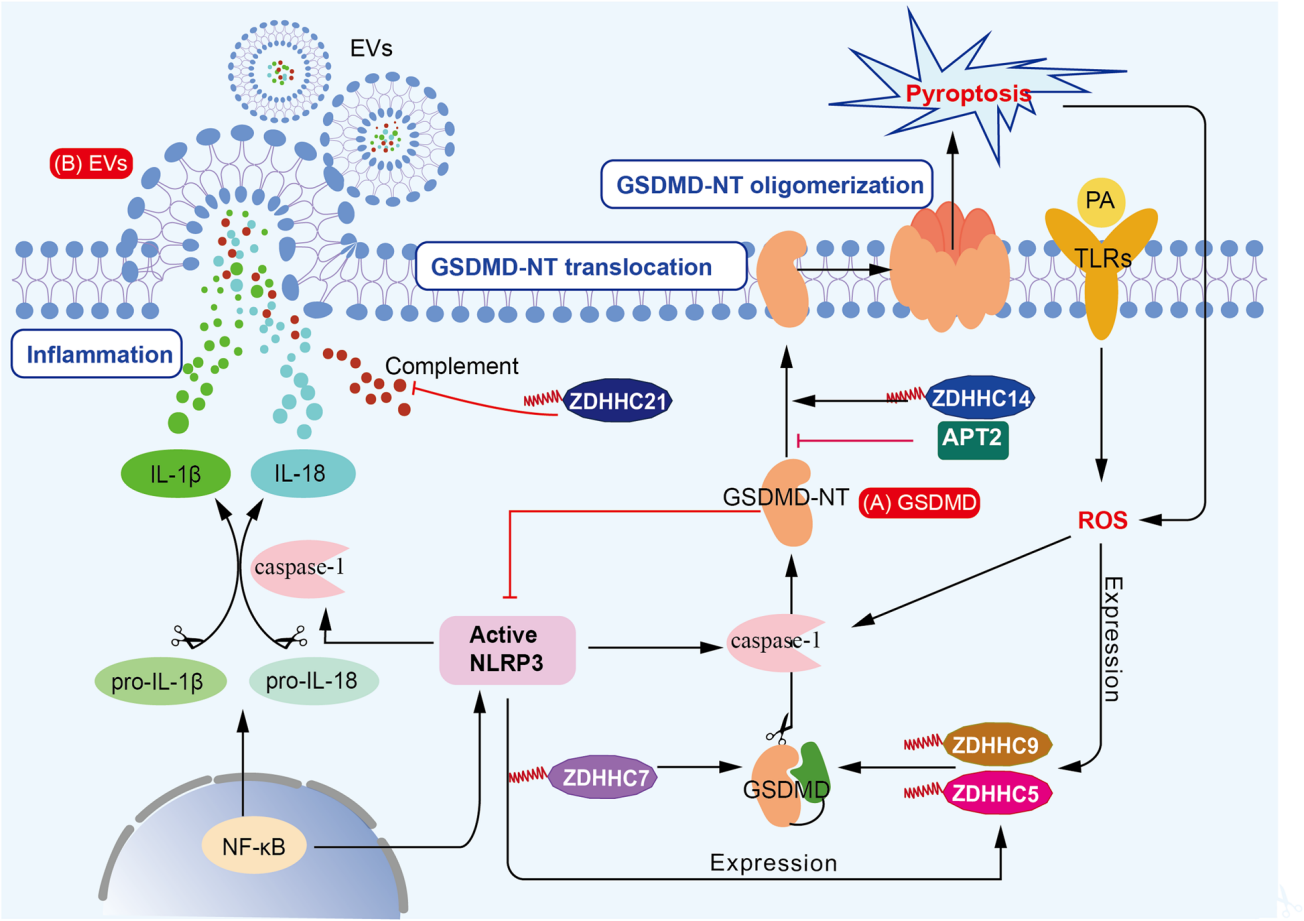
Relationships between palmitoylation and sepsis-related key factors (GSDMD, EVs). **A** Active NLRP3 induces caspase-1 to cleave pro-IL-1β and pro-IL-18 into their mature forms, IL-1β and IL-18, thereby promoting inflammation. Concurrently, caspase-1 cleaves GSDMD into GSDMD-NT, a process enhanced by ZDHHC7 (Zhang et al. 2024). GSDMD-NT exhibits membrane affinity and undergoes oligomerization to induce pyroptosis. ZDHHC14 facilitates its membrane translocation, wherase APT2 reverses this process (Zhuang et al. 2024). The combination of PA and TLRs increases cytoplasmic ROS (Nicholas et al. 2017; Du et al. 2024). Elevated ROS and active NLRP3 both promote the expression of ZDHHC5 and ZDHHC9, which further enhance GSDMD cleavage by caspase-1 (Du et al. 2024). **B** Cytokines are encapsulated into vesicles, which are then released as EVs via exocytosis. Similarly, the formation of complement-associated EVs is enhanced through ZDHHC21-mediated palmitoylation (Yang et al. 2021). Abbreviations: APT, acyl-protein thioesterase; EVs, extracellular vesicles; GSDMD, Gasdermin D; GSDMD-NT, Gasdermin D N-terminal fragment; PA, palmitic acid; ROS, reactive oxygen species; TLRs, Toll-like receptors

GSDMD is a key executor of cell pyroptosis. Activated caspase-1 cleaves GSDMD into GSDMD-NT, which forms pores in plasma membrane, ultimately contributing to cell pyroptosis during sepsis development. In this process, the palmitoylation of GSDMD by ZDHHC7 at Cys192 directs its cleavage by caspase promoting its activation (Zhang et al. 2024). Palmitoylation of GSDMD-NT enhances its translocation to the plasma membrane, promoting its oligomerization and amplifying its pyroptotic activity, which APT2 depalmitoylates it (Balasubramanian 2024). GSDMD-mediated pyroptosis also dependents on ROS stimulation. Furthermore, its level in the cytoplasm can be escalated through the synergistic action of PA and TLRs, and activation of pyroptosis (Nicholas et al. 2017; Du et al. 2024). Moreover, palmitoylation can enhance GSDMD activation which is further augmented by ROS signaling (Du et al. 2024). ZDHHC5 and ZDHHC9 are major palmitoyltransferases for GSDMD, driving cell death and promoting pro-inflammatory events. Their expression is upregulated by inflammasome activation and ROS (Du et al. 2024). Thus, we can reasonably predict a preliminary positive feedback model related to the inflammatory response and pyroptosis under S-palmitoylation modification. In contrast, GSDMD-NT has been shown to act as a negative feedback regulator, controlling inflammasome activity (Hu et al. 2022). Whether S-palmitoylation directly participates in this negative feedback is still unclear. More pervasively, researchers have proposed a two-step mechanism for GSDMD oligomerization: initial palmitoylation at Cys192, followed by subsequent palmitoylation at Cys39, Cys57, and Cys192 (Margheritis et al. 2024). This model indicates that the regulation of protein S-palmitoylation is orchestrated both spatially and temporally, with the effects of each biological step being cumulative. Strategically, inhibiting key palmitoylation-related processes involved in GSDMD-induced macrophages pyroptosis and IL-1β release could attenuate sepsis-induced organ injury. In mice with acute myocardial infarction (AMI), disulfiram was found to antagonize ZDHHC14-induced palmitoylation at Cys192, interfering with GSDMD localization to the cytomembrane and reducing myocardial pyroptosis and injury (Zhuang et al. 2024). Another novel GSDMD inhibitor, NU6300, interacts with Cys191, impairing palmitoylation of the full-length GSDMD and GSDMD-NT, suggesting its extensive effects in sepsis treatment (Jiang 2024). However, the underlying crosstalk between these promosing drugs and sepsis-induced inflammatory and immunosuppressive microenvironment, including ZDHHC enzymes involved and the individual or combined residues palmitoylated, remains to be fully exploited and systematically designed. It is anticipated that the discovered palmitoylation sites of GSDMD could serve as promising therapeutic targets. We described the palmitoylation impacts on GSDMD in sepsis. The relevant content is exhibited in Fig. 3.

#### STING

STING is typically localized in ER. After cGAS synthase, an cytosolic DNA sensor, combines with exogenous DNA, it produces cyclic GMP-AMP (cGAMP), which activates STING. Activated STING then recruits TANK-binding kinase 1 (TBK1), leading to phosphorylation of IRF3 and activation of NF-κB, ultimately inducing the expression of type I IFN, TNF, and IL-6 (Cheng 2020). Thrombosis and excessive neutrophil activation are major contributors to multiple organ dysfunction caused by sepsis. Notably, STING has been associated with platelet activation and NET formation, both of which can be regulated by palmitoylation, implying its meaning in MODS (Yang et al. 2023). Additionally, cGAMP can enhances STING palmitoylation in platelets (Zhang et al. 2021). However, whether cGAMP is necessary for STING palmitoylation remains unclear. Interestingly, using scRNA-seq, MS, and other genetic techniques, researchers found that STING can induce macrophage ferroptosis independently of cGAS and IFN, thereby contributing to septic organ injury (Wu et al. 2022). The findings suggest the existence of additional pathways that may regulate STING activation. Coincidentally, in macrophages, FASN-dependent palmitoylation of STING at Cys91 residue can alleviate sepsis-induced liver injury, revealing a novel paradigm of STING regulation (Kang et al. 2024). Meanwhile, palmitoylation can exert opposing effects on the cGAS/STING pathway in infectious diseases. For instance, palmitoylation of cGAS at Cys474 reduces its enzymatic activity in the presence of double-stranded DNA, mainly by inhibiting DNA virus interaction and disrupting cGAS dimerization (Shi et al. 2022). The inhibitory role of palmitoyltransferase ZDHHC18 on cGAS has been demonstrated in this process (Shi et al. 2022). In addition, researchers have attempted to explore drugs targeting STING palmitoylation. For example, a recent STING inhibitor, indolyl-urea, was published to block STING palmitoylation and subsequent signalling transduction, suggesting that palmitoylation regulators may serves as potential therapeutics for sepsis (Han et al. 2025). Analogously, 4-octyl itaconate restricts STING activation by preventing its palmitoylation and interacting with alkylation (Zhuang 2025). In summary, these findings underscore the dual regulatory role of palmitoylation on cGAS/STING pathway. However, the detailed palmitoylating effects on STING activation in sepsis remain largely unexplored. The palmitoylation influences on STING signaling are shown in Fig. 2.

#### Other

In addition to palmitoylation of common sepsis-related effectors, extracellular vesicle (EVs) represent a typical form of intercellular communication and have been studied for decades. EVs are released from cells during activation or apoptosis and carry membrane epitopes specific to their parental cells, underscoring their heterogeneity in various studies. During the acute stage of sepsis, EVs derived from macrophages, neutrophils, monocytes, and platelets function as carriers of pro-inflammatory cytokines and regulators of cell differentiation and activation (Wang et al. 2021; Raeven et al. 2018). The complement-related pathway plays a key role in mediating their activation of innate immunity. EVs also contribute to the transport of complement components. Studies have confirmed that sepsis significantly increases the number of EVs, along with a marked rise in cellular palmitoylation levels (Yang et al. 2022). Later, researchers found that depletion of ZDHHC21, a PATs mainly located on the plasma membrane, reduces complement components in EVs and diminishes their ability to activate neutrophils (Xie et al. 2021). In ZDHHC21-depleted mice, the number of neutrophils aggregated in lung significantly decreased, further highlighting ZDHHC21’s role in modulating immune responses during sepsis (Yang et al. 2021). Therefore, treatments targeting EVs have been proposed. For example, designed vesicles enriched with palmitoylated-ACE2 have been developed as a potential therapy against COVID-19, demonstrating the therapeutic versatility of EVs (Xie et al. 2021). Beyond the EV-associated palmitoylation in sepsis, this modification also contributes to other aspects of sepsis progression. ZDHHC18 and ZDHHC21, two palmitoyltransferases, are both up-regulated in CLP-induced renal injury, mainly palmitoylating α1 AR to sustain its responsiveness to phenylephrine (Marin et al. 2016). This sustained receptor activation may exacerbate renal dysfunction by amplifying stress response in the kidneys. During sepsis-induced liver injury, LPS upregulates CD36 expression and facilitate the formation of CD36-UBQLN1-SNARE complex. This complex obstacles lysosome and autophagosome synthesis, leading to hepatocyte injury, which this process is positively regulated by CD36 depalmitoylation (Li et al. 2023). Additionally, S-palmitoylation of TNF-α regulates its interaction with TNFR1 by altering TNF’s lipid raft partitioning (Poggi et al. 2013). These observations highlight the multifaceted modulatory modes of palmitoylation across various receptors in sepsis. Given its versatility and context-dependent effects, defining the precise molecular context in sepsis crucial for exploring. The EVs-related palmitoylation is described in Fig. 3.

### Perspectives for the future

As previously discussed, the regulation of palmitoylation and depalmitoylation plays a universal role in processes such as cargo sorting, protein localization, stability, and signal transduction. Together, they from a vast post-translational regulatory network that orchestrates the spatial and temporal distribution of exogenous or endogenous agents, playing a crucial role in maintaining homeostasis and contributing to pathological dysfunction. Understanding the dynamics of palmitoylation and depalmitoylation has a significant impact on elucidating mechanisms in sepsis progression and developing novel therapies for septic patients.

A review of sepsis-related studies reveals that, beyond its broad regulatory effects, several notable features may also be associated with this modification. Firstly, palmitoylation is a dynamic process with temporal and spatial specificity, primarily reflected in the sequential cycles of palmitoylation and depalmitoylation that activate or dysregulate target moleculars or pathways. Additionally, the transport of palmitoylated molecules between cells and organelles leads to distinct functional outcomes. Given these properties, it is highly valuable to investigate the effects of this dynamic regulation in sepsis, especially in the hyperinflammatory early stages and the immunosuppressive later stages. Whilst, most of recent studies related to palmitoylation and sepsis are usually associated with hyperinflammation, which inspired us what kind of palmitoylation features will be in immunosuppressive circumstances. Protein modifications in different immune cells and tissues affected by sepsis warrant systematic investigation. Secondly, palmitoylation at different sites within a single molecule may have additive effects, providing diverse regulatory possibilities. These additive effects collectively enhance signal transduction. However, whether palmitoylation at different sites exhibits mutual exclusivity remains unknown. It is clear that identifying palmitoylated proteins through proteomics may be insufficient. The development of advanced technologies is essential to expand the scope of palmitoylation research and clarify the functional roles of multisite modifications. Thirdly, this widespread post-translational modification involves both positive and negative feedback loops, aligning with biological progression and adaptation. These feedback mechanisms help maintain systematic balance and provide a framework for understanding how palmitoylation contributes to pathophysiological changes during sepsis.

Despite notable progress, significant challenges remain in elucidating the detailed mechanisms of palmitoylation in sepsis. Firstly, although inhibitors for APTs, PPTs, and ABHDs exist, their efficacy in vivo and vitro remains poorly validated. Furthermore, the lack of specific ZDHHC inhibitors, robust palmitoyl-mimetic mutations, and defined consensus palmitoylation sequences hampers the advancement of gain-of-function studies. Additionally, emerging palm-proteomic techniques may yield false-positive results. To improve accuracy, it is recommended to combine proteomic methods with ABE, click chemistry, and point-mutagenesis to reliably measure palmitoylating levels, identify target proteins, and determine their functions. Elucidating the underlying mechanisms of palmitoylation in sepsis requires further effort.

## Conclusion

In summary, the role of palmitoylation in the development and regulation of sepsis is becoming increasingly clear. Recent studies have highlighted its involvement in various pathways and cellular processes to sepsis, particularly inflammation and multiple organs dysfunction. Furthermore, palmitoylation-related characteristic were summarized in our review, offering inspiration and promising directions for future sepsis researche. However, exploring the precise mechanisms underlying palmitoylation and sepsis remains a major challenge, requiring advanced technologies and extensive systematic expermentation.

## Data Availability

No datasets were generated or analysed during the current study.
